# Model-Based Prediction of the Tensile Properties of Polypropylene Films Made from Recycled Materials

**DOI:** 10.3390/polym17081044

**Published:** 2025-04-12

**Authors:** Marius Stieglitz, Sonja Adomeit, Marisa Müller, Karina Hettwer, Anja Schlierf, Steffen Uhlig, Sarah David, Christian Hopmann, Lisa Leuchtenberger-Engel

**Affiliations:** 1 Institute for Plastic Processing (IKV) in Industry and Craft at RWTH Aachen University, Seffenter Weg 201, 52074 Aachen, Germany; 2 Ehrenmüller GmbH, An der Stadtmauer 4, 87435 Kempten, Germany; 3 quo data Gesellschaft für Qualitätsmanagement und Statistik mbH, Prellerstraße 14, 01309 Dresden, Germany

**Keywords:** flat film, mechanical properties, material properties, recyclate, modeling

## Abstract

This study investigates the potential of data-driven modeling to monitor and predict mechanical properties of extruded film using recyclates. The first step is to investigate whether a process parameter can indicate the input material quality of the material, which can vary significantly due to the use of recyclate. The die pressure was shown to be a key indicator due to its strong correlation with viscosity and material degradation. The second step is to explore the ability of machine learning models—Generalized Additive Models, Linear Regression, and Random Forest—to predict film tensile strength and modulus based on extrusion process and material parameters. The results demonstrate that including Melt Flow Rate and shear viscosity in addition to pressure and feedstock type (Virgin, PIR, and PCR) significantly improves model accuracy, with Generalized Additive Models achieving the highest R^2^ of 85.7% for tensile strength prediction. Additionally, the observed variability between different recyclate streams highlights the need for a more detailed classification of recyclates to better predict and optimize the mechanical performance of the film. While data-driven approaches for predicting properties show promise, their effectiveness remains limited by data availability and feedstock variability. Expanding datasets and improving process stability will be critical to refining predictive models for industrial application.

## 1. Introduction

In 2023, 413.8 billion tons of plastics were produced worldwide [1]. This enormous amount shows the relevance of plastics technology in the global economy. It is therefore important to promote the sustainable production of this material. However, achieving comprehensive sustainability in plastics manufacturing is complex, with various challenges limiting the current global utilization of post-consumer recyclates (PCRs) to a mere 8.7% of total production volume [1]. The reasons why the use of PCRs is inhibited include unavailability, quality loss, and the cost structure [2,3,4].

Nevertheless, the proportion of PCRs used in Europe has increased by 70% since 2018, partly due to legal requirements and guidelines like the Packaging and Packaging Waste Regulation [5,6]. This is of immense importance, as plastics contribute to increased productivity and efficiency in the area of application and product shelf life in a wide range of applications, such as agriculture and food packaging.

The aforementioned quality of PCRs is a challenge for companies in production, such as flat film extrusion. On the one hand, seasonal fluctuations in feedstock material result in variability in quality throughout the year and, on the other hand, the material quality decreases due to a lack of varietal purity and degradation [7,8,9]. As a result, the product, e.g., packaging film, has an unpleasant odor, a deteriorated appearance, and altered mechanical properties [10,11,12].

In the packaging sector, thermoforming is a widely used manufacturing process that plays a major role in the production of cups, trays, and blisters. The great freedom of design offers a wide variety of products. Polypropylene is the most commonly used semi-finished product in thermoforming. However, a high level of process knowledge is already required when processing virgin PP, meaning that a high-quality film is essential as an input material [13].

To face these challenges, it is essential to monitor the process and product quality with established measurement technologies to detect deviations in the film extrusion process under the use of PCRs. Due to the exponential increase in digitalization and the use of artificial intelligence in recent years [14], there is immense potential to further develop the measurement technology method and to expand it to include the prediction of a wide range of film characteristics, including, among others, tensile strength and modulus.

## 2. State of the Research Field

### 2.1. Plastic Recyclates

The PCR properties must meet the specifications to ensure uniform film properties over the entire process and the utilization phase [15]. However, it must be considered that recyclates are exposed to various thermal, mechanical, and chemical loads during their use and processing, which can lead to damage to the polymers and potentially negative effects on the subsequent product properties [16].

#### 2.1.1. Different Types of Mechanically Recycled Material

There are generally two types of mechanically recycled materials: post-industrial recyclates (PIRs) and PCRs [17]. Post-industrial recycling comprises the utilization of plastic waste generated during the industrial processing of virgin plastic. The surplus and, by the previous extrusion step, slightly degraded material is collected, mixed with other virgin plastic, and fed back into the production process. The return of edge trimmings from the films and the waste after cutting out thermoformed parts is in many cases a very suitable material flow [18]. PIRs are very clean and usually single-origin, and, in most cases, enable conventional production. PCRs, on the other hand, are reprocessed plastics from household or commercial waste, the majority of which is packaging. A distinction can be made between these materials based on the feedstock, with virgin materials being classified as primary raw materials, PIRs as secondary raw materials, and PCRs as tertiary raw materials.

#### 2.1.2. Degradation of Plastics

Degradation occurs both during processing and during the utilization phase [19]. These mechanisms are caused by time- and temperature-dependent changes of the material, environmental conditions, mechanical stress, and the degree of exposure to light [16,20,21,22,23]. In general, plastics are only stabilized for processing and the first life cycle, but not for further processing or longer application times. This problem is particularly relevant for plastic films, which are made of polyolefins, such as PP and PE, for packaging purposes and generally have a short service life [16].

During processing or extrusion, plastics are subjected to processing temperatures and shear forces [24]. As a result, cross-links and chain branching are formed [25]. The resulting molecular damage leads to changes in molecular weight and molecular weight distribution, which in turn causes a change in viscosity and a reduction in optical and mechanical properties compared to virgin material [15]. Besides polymer degrading, contaminants, e.g., printing ink and other chemicals, also affect the quality of the recycled material, and their influence on the preparation and processing process varies depending on their size and quantity.

During the recycling process, the rheological, optical, and mechanical properties of the plastic change, making it necessary to adapt the process conditions in order to avoid difficulties during processing. Compared to virgin material, recycled products may also have changed mechanical properties. Due to the complex relationships between process conditions and the resulting film characteristics during processing and the additional influence of the recyclate, the properties of the resulting film products can only be predicted offline in time- and resource-consuming procedures, such as analytical measurements.

### 2.2. Modeling in Material Science and Plastics Processing

The field of materials science is increasingly utilizing machine learning (ML) to accelerate the discovery and development of new materials, with a growing focus on incorporating recycled components, moving away from traditional trial-and-error methods. Chong et al. [26] provide a comprehensive overview of ML methods and their applications in materials science, highlighting the potential of these techniques to rapidly screen and generate materials with the desired properties.

This shift towards ML-driven approaches is evident in various subfields of materials science. For instance, Lopez-Garcia et al. [27] focused on applying artificial intelligence to optimize the re-manufacturing of recycled fibers. They developed a system trained on experimental data to predict parameters for compounding recycled fibers, enabling the efficient production of recycled fiber composites with targeted material properties. Salehi et al. [28] used various ML models to predict the rheological properties of recycled plastic modified bitumen. The CatBoost model performed best, achieving R^2^ values of 98% for stiffness and 93% for elasticity predictions. Key factors influencing the recycled plastic modified bitumen performance were identified as the temperature, frequency of testing, amount of polymer, and type of bitumen. In the realm of polymer composites, Seifert et al. [29] developed an analytical model to predict the tensile modulus of polypropylene compounds containing various additives and fillers, achieving a coefficient of determination R^2^ of 98%. This model proved effective in optimizing compound formulations and reducing the need for extensive experimental testing. In that study, however, only injection moulding compounds were processed and optimized, and there is no reference to film types. Similarly, Altarazi et al. [30] employed ML algorithms (e.g., k-nearest neighbor and support vector machine) to predict the tensile strength of polymer films produced using, e.g., virgin and recycled high-density polyethylene. Their study demonstrated the effectiveness of a support vector machine algorithm in predicting tensile strength, achieving a high degree of accuracy (R^2^ = 96%); however, unlike the present study, they did not investigate the influence of recycled materials. These studies exemplify the growing potential of ML in optimizing material design and processing, paving the way for more efficient and sustainable manufacturing practices. Nevertheless, a significant gap remains in the understanding of how PCR materials influence film properties, hindering progress towards circularity in this sector and demanding further research.

## 3. Objective and Scope of the Work

The main objective of this work is the modeling of the mechanical properties, namely tensile modulus and strength, of an extruded film, particularly for blends of recyclates with virgin material. The use of recyclates and their fluctuating input qualities are a major challenge for manufacturers and require a model-based analysis of the process to monitor and predict the properties. Therefore, this study pursued two research objectives:

1. Is there a set of process parameters that can indicate the input material properties in the PP film extrusion process?
2. Can ML models predict the resulting film tensile modulus and strength using these process parameters?

To answer these questions, various flat films were produced. Blends of various recyclates with virgin material were used as feedstock material, and the films were subsequently characterized for their mechanical properties in a tensile test. By recording the entire process data of the extrusion line (die pressure, barrel and melt temperatures, screw speed, and torque), a set of process parameters was selected from the data that is suitable for modeling the mechanical properties of the extruded film. Eventually, ML models were employed to predict the tensile modulus and strength of the final film product. This predictive capability facilitates improved process control and optimization in flat film extrusion when utilizing recycled materials.

## 4. Materials and Methods

### 4.1. Flat Film Extrusion Line

Production of the flat film was carried out on a laboratory extruder (screw diameter D = 19 mm, length of the three-zone screw of 25 D) and a calendar type 136–350, both from Collin Lab & Pilot Solutions GmbH, Maitenbeth, Germany. For the melt discharge, a monofilm die with coat hanger distributor from Brabender GmbH & Co. KG, Duisburg, Germany, was used (exit gap width = 150 mm, exit gap height = 0.2–1.2 mm). A motor with integrated speed and torque measurement was installed for the data acquisition. The torque was calculated via the applied motor current. A pressure sensor (Measuring range 0–50 bar—Accuracy 0.5%) from Gneuss, Bad Oeynhausen, Germany, was used to record the melt pressure before the die [31]. Pressure is frequently measured in industrial applications, while the melt temperature is rarely measured, especially with immersion sensors. For this reason, analysis of a temperature sensor was not considered in this study.

### 4.2. Materials

Six different PP materials were used to extrude flat films. Among them was one virgin material (Moplen HP501M—LyondellBasell Industries N.V., Rotterdam, The Netherlands), named as Virgin for the further course of the study, two PIRs, and three PCRs from various suppliers. The recyclates were mixed with the virgin material in various blends (see Section 4.4), as this is a common practice in industrial applications, especially for mono-material films. To characterize the flow properties of the unmixed components, the Melt Flow Rate (MFR—DIN EN ISO 1133-1 [32]—230°/2.16 kg) as a typical data sheet parameter was measured, and the shear viscosity was measured for different shear rates at 220 °C. The gradient of the linear fit was determined from the three shear viscosity values and used as an input parameter for modeling. The characteristic values of the materials can be found in Table 1. The PIR material was an extrusion grade, which results in a lower MFR. The virgin material and the PCR materials were injection molding grades with a higher MFR. Detailed pressure data are provided in Appendix A.

### 4.3. Determination of the Mechanical Properties

**Figure 1:**
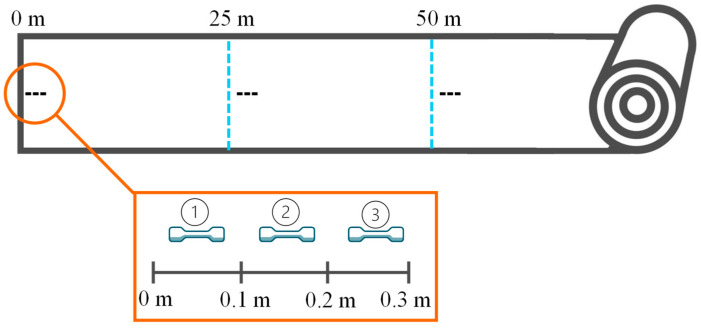
Sampling and measuring position on the produced film rolls.

The mechanical properties of a film are among the most important performance indicators and appear in every requirement profile and technical data sheet. A high tensile strength is particularly important for packaging. To quantify the mechanical strength, tensile tests were carried out in accordance with DIN EN ISO 527-3 [33], using a Zwick Z10 universal testing machine from ZwickRoell GmbH & Co KG, Ulm, Germany. Nine specimens were tested per test point along the film roll over a total of 50 m (see Figure 1). Rectangular specimens of type 2 with a width of 15 mm were tested [33].

### 4.4. Experimental Plan

The test series maintained constant process parameters (Table 2), varying only the ratios of virgin and recycled materials in the blend. This controlled approach allowed for direct analysis of how different material compositions influenced the properties of the final film. The test plan is shown in Table 2. It should be noted that test IDs 1–17 and 18–28 were carried out in two separate test series.

### 4.5. Data Analysis

#### 4.5.1. Data

The dataset consists of 28 tests of either virgin only or different mixing ratios between virgin material and recyclates (see Table 2). The mechanical properties defined as target values to answer the research question were tensile modulus and tensile strength. For both properties, three specimens were taken at three different foil sections, resulting in nine measurements per test (see Figure 1). The following parameters were considered: melt pressure, torque, feedstock, MFR, and the gradient of shear viscosities (see Table 3). The statistical modeling and evaluations were performed in R [34] and Python 3 [35] programming languages.

#### 4.5.2. Machine Learning

Traditional ML algorithms, such as Generalized Additive Models (GAMs), Linear Regression (LR), and Random Forests (RF) [36] were employed, as owing to the relatively small dataset size, advanced deep learning models were deemed inappropriate. ML algorithms learn from data without explicit programming. Supervised learning, a key paradigm, involves training models on labeled data to predict outcomes for new inputs.

LR [37,38,39] is a statistical method for modeling the relationship between a dependent variable and one or more independent variables. It assumes a linear relationship and attempts to find the best fitting line through the data points. This line is represented by a linear equation in which the coefficients are estimated to minimize the sum of squared errors between the predicted and actual values.

GAMs extend conventional linear models by allowing the linear predictor to depend linearly on unknown smooth functions of predictor variables, while maintaining the additive model structure. This flexibility enables GAMs to capture complex, nonlinear relationships between the predictors and the response variable, making them highly suitable for a wide array of real-world applications where interactions are not strictly linear [40,41].

Each smooth function is typically represented using basis splines, smoothing splines, or other non-parametric techniques, which are estimated through penalized likelihood maximization to avoid overfitting and ensure model robustness. This penalization process involves selecting an appropriate level of smoothness for each function, balancing the trade-off between fit and generalization. By employing techniques such as cross-validation (CV) (see Section 4.5.4), GAMs automatically adjust the degree of smoothness, tailoring the model complexity to the intrinsic nature of the data [40,41].

RF is an ensemble learning method that combines multiple decision trees to improve prediction accuracy. Each tree in the forest is trained on a random subset of data and features. By combining the predictions of these individual trees, such as by averaging them, RF can reduce overfitting and improve generalization performance [37].

By using ML models to predict the tensile modulus and strength of the final film, we aimed to optimize and control the flat film extrusion process when recycled materials are used.

#### 4.5.3. Data Preprocessing

The average pressure at the corresponding time of sampling was computed as the pressure input. The feedstock was either ‘Virgin’, ‘PIR’, or ‘PCR’ for mixtures and was converted to a numerical representation specific to the model. For the MFR and shear viscosity values, the weighted average was calculated considering the proportions of different polymer types in the mixture. Shear viscosity was not directly input but inferred from the gradient of a linear regression line through the three shear rates (see Table 1). In total, *n* = 252 data points for tensile modulus, and *n* = 247 data points for tensile strength were available after outlier exclusion. For the GAMs, all available measurements were considered. For the LR and RF models, the target values for both tensile modulus and strength were derived by taking the mean of the three measurement points at each film section, resulting in a final dataset size of *n* = 84.

For modeling purposes, three sets of input features were constructed. Feature set (FS) 0 used only the pressure from the film extrusion process and feedstock. Feature set 0 was then extended by including the MFR, meaning that feature set 1 included pressure, feedstock, and MFR. The MFR value is typically readily available with minimal effort, as it is provided by the polymer manufacturer/recycler in the technical datasheet. Feature set 2 further extended feature set 1 by including the viscosity gradient. Table 4 provides an overview of the composition of the feature sets.

For the modeling, the melt pressure and mechanical properties were normalized to virgin PP. This was done to address considerable differences in melt pressure observed across different experiments. For the melt pressure, the mean absolute deviation between both test series for Virgin and blends of PIR 2 and PCR 2 (share of 50%) with Virgin were −1.5, 1.5, and −1 bar. The related mean relative deviations between test series were −18%, 15% and −14%. These deviations can be attributed to the measurement setup and are discussed in more detail in Section 6.

To enhance the predictive power of the later fitted LR, we employed a polynomial transformation of the input features MFR, pressure, and the viscosity gradient. This transformation introduced new features: MFR squared, pressure squared, as well as the interaction terms between MFR and pressure and viscosity gradient and pressure, allowing the model to capture potentially nonlinear relationships between the features and the target variable.

#### 4.5.4. Evaluation

The Pearson correlation coefficients (r) [42] were calculated to assess the relationship between the target properties of tensile strength and tensile modulus with the designated model inputs, the extrusion pressure, the MFR, and the viscosity gradient. r is a statistical metric measuring the linear relationship between two variables, ranging from −1 (perfect negative correlation) to 1 (perfect positive correlation). Values closer to −1 or 1 indicate stronger relationships, while values closer to 0 suggest weaker or no relationships. It is essential to note that a strong correlation between two variables does not necessarily indicate a causal relationship between them.

CV is a ML technique that evaluates a model’s ability to generalize to unseen data [43]. It mitigates overfitting, where a model excels on training but performs poorly on new data, particularly prevalent in scenarios with limited data availability.

The data was divided into multiple subsets. One subset acted as a test set while the model trained on the remaining subsets. This process was repeated, with each subset serving as the test set once. By averaging the performance across iterations, CV provides a robust estimate of the model’s true predictive performance on unseen data.

To rigorously evaluate the performance of the developed models, a suite of evaluation metrics was employed. These metrics were computed for each subset during the CV process and subsequently averaged. The mean error (ME) provides a central tendency measure, denoting the bias. While ME can be positive or negative, reflecting over- or under-prediction, the mean absolute error (MAE) measures the average magnitude of errors regardless of their direction. The standard deviation (SD) of the error quantifies the dispersion of errors around the mean. Furthermore, we calculated the mean absolute percentage error (MAPE), root mean squared error (RMSE), and objective function value (OBJ) [44], as well as the coefficient of determination, R^2^ [44]. These metrics collectively offer a comprehensive evaluation of the model‘s accuracy, bias, and variability in predicting the target variable (see Appendix B).

## 5. Results

For the model-based prediction of the mechanical properties of a film made from recycled material, a process parameter that is representative of the input material properties was determined in a first step. This can be used in combination with material characteristics to determine a correlation analysis which will form the basis for training various ML algorithms.

### 5.1. Determination of a Characteristic Process Key Value

To address the research objective “Is there a process parameter that can monitor the input and output quality in the film extrusion process?”, the torque applied to the screw and the resulting die pressure were investigated. These two variables are linked to each other, but a process parameter was searched for that has low measurement fluctuations and can indicate the input material quality. Recycled materials show higher variation than virgin materials, due to the reasons mentioned in Section 2.1.2. Materials of varying viscosities exhibit divergent behaviors during the extrusion process, emphasizing the necessity for precise adjustment of process parameters during the extrusion of recycled materials [45].

Consequently, quality monitoring during the extrusion process has become mandatory, as poor mechanical properties of the product can be easily overlooked during the final control stage. The ability to regulate melt temperature, melt pressure, and viscosity is often directly related to achieving acceptable quality. In general, the melt viscosity and the distribution of melt temperature across the melt flow are considered key indicators of melt quality in polymer extrusion processes [46]. There have been several strategies developed to implement the key process values’ in-line monitoring. The approach focuses on viscosity measurement as a control parameter, as polymer viscosity correlates with its composition and molecular distribution [47]. In addition to in-line rheometers [48] and models of viscosity in the process [49,50] die pressure and torque, measured variables recorded in every extrusion line, were selected as characteristic values. Die pressure and torque are correlated with viscosity as follows.

(1) $$\Delta p=\frac{12\dot{V}L\eta_{newtonian}}{B\;*\;H^{3}}$$

(2) $$M_{d}=\frac{P}{2\pi n_{0}}$$

(3) $$P=\tau \left(\eta \right)\;*\;v_{circ}$$

Thus, according to Equation (1), the pressure Δp is dependent on the volume flow $\dot{V}$ and the geometry parameters L, *B*, and *H* of the die. With increasing viscosity of the plastic, and all other parameters remaining the same, the pressure also increases. The torque applied to the screw is calculated according to Equation (2) using the power *P* and the speed of the screw *n*_0_. The power P is in turn calculated using the circumferential speed $v_{circ}$ and the viscosity-dependent shear stress $\tau \left(\eta \right)$ (Equation (3). An increasing viscosity is accompanied by an increasing torque.

**Figure 2:**
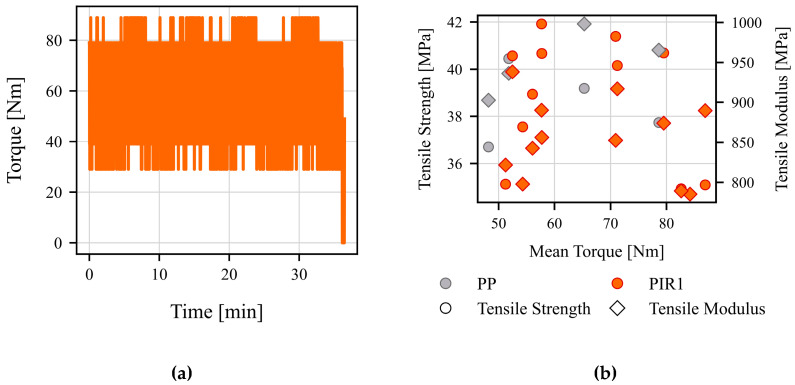
Exemplary visualization of the torque recorded in a preliminary test, not covered in this paper: (**a**) exemplary torque progression of 100% PIR1; (**b**) relationship between torque and the target variables tensile strength and modulus.

**Figure 3:**
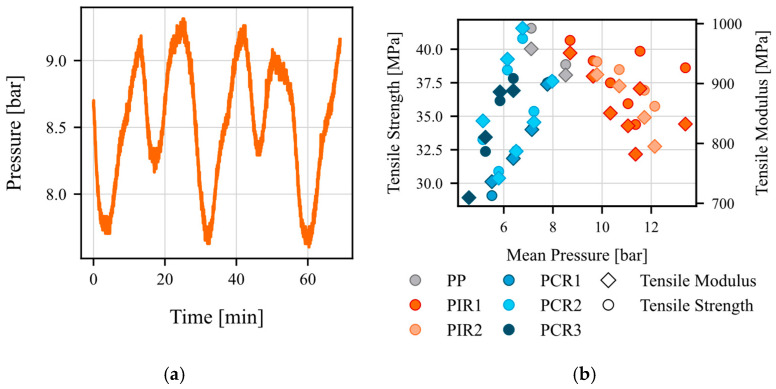
Visualization of melt pressure: (**a**) exemplary pressure progression for T01 of 100% virgin material; (**b**) relationship between melt pressure and the target variables tensile strength and modulus.

An analysis of the torque (Nm) during the extrusion process revealed significant variability and inconsistency, mostly ranging between 25 Nm and 85 Nm (see Figure 2a). No clear relationship was observed between the torque and the properties of interest, tensile strength and modulus (see Figure 2b). These high fluctuations in the torque are due to the measurement method. In the method used, the torque is calculated via the motor current and as can be seen, is subject to very high fluctuations due to the electrical connection. A better method would be to use a direct measuring flange on the shaft of the screw, which would lead to a more accurate measurement of the torque. In contrast, melt pressure (bar) exhibited more stable behavior and a strong correlation with both target parameters (see Figure 3). The cyclical fluctuations in the pressure measurement can be attributed to a variety of causes and complex interactions. These include the material itself, the screw design, the operating point, and the solids behavior. The fluctuations that occur are small and can be compensated for by averaging. Consequently, melt pressure was selected as the preferred input parameter for the subsequent model development.

### 5.2. Correlation Analysis Between Process Parameters, Material Characteristics, and Mechanical Film Characteristics

The correlation analysis revealed distinct trends between the input parameters and material properties for PCR and PIR. For PCR, the MFR showed strong negative correlations with both tensile modulus (r = −0.78) and strength (r = −0.82) (see Table 5). Conversely, the PIR materials exhibited a strong positive correlation between MFR and tensile modulus (r = 0.9). Pressure demonstrated a strong positive correlation with tensile properties for PCR (r = 0.91 for modulus, r = 0.94 for strength), notably stronger than for PIR (r = −0.66 and r = −0.56, respectively). For PCR, the viscosity gradient showed a negative correlation with tensile properties (r = −0.70 for modulus, r = −0.73 for strength). PIR1 exhibited strong positive correlations between viscosity gradient and tensile properties (r = 0.93 for modulus, r = 0.92 for strength), while PIR2 displayed strong negative correlations (r = −0.91 and r = −0.90, respectively). Overall, the analysis between the input parameters and material properties highlights opposing relationships for PCR and PIR.

**Figure 4:**
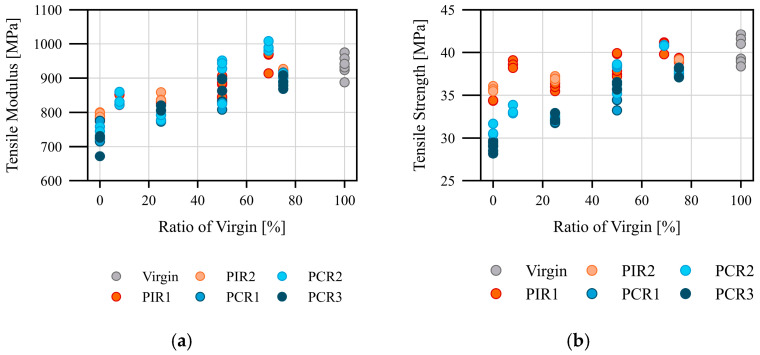
Mechanical characteristic values for the film with increasing proportion of virgin material/PP: (**a**) tensile modulus; (**b**) tensile strength.

In Figure 4a tensile modulus and in Figure 4b tensile strength are plotted against the proportion of virgin material with the different feedstocks. In addition to the, in some observations, high deviation of the test points within a material and variability between the feedstocks, there is a linear trend towards higher mechanical properties with the addition of virgin material/PP. This correlation was expected as, according to the recyclate manufacturers’ data sheets, the tensile strength and tensile modulus are lower than those of virgin material [51]. Furthermore, the importance of the material quality of the recyclate can be seen in PCR2, which achieved the highest mechanical values in almost every mixing ratio compared to PCR1 and PCR3.

### 5.3. Modeling and Prediction of Mechanical Film Characteristics Based on Process Parameters and Material Characteristics by Means of ML Algorithms

To answer research question 2 (Can ML models predict the resulting film properties using this process parameter?), the feedstock type, the process parameter melt pressure, the material characteristics, the MFR, and the viscosity gradient were considered as input features for modeling. A set of different ML models was fitted, and the model performance was evaluated.

Initially, all data points were included, and GAMs were fitted to investigate if melt pressure is a suitable process parameter to predict mechanical properties. Subsequently, material properties, particularly MFR and the viscosity gradient, were added to the model to assess if the model performance increased.

For the GAMs using all data (see Table 6), adding MFR and the viscosity gradient significantly improved the performance compared to the baseline scenario with feature set 0. For the tensile modulus, the R^2^ values were 78.70%, 83.27%, and 86.79%, and for tensile strength, they were 91.56%, 94.74%, and 96.91%.

**Figure 5:**
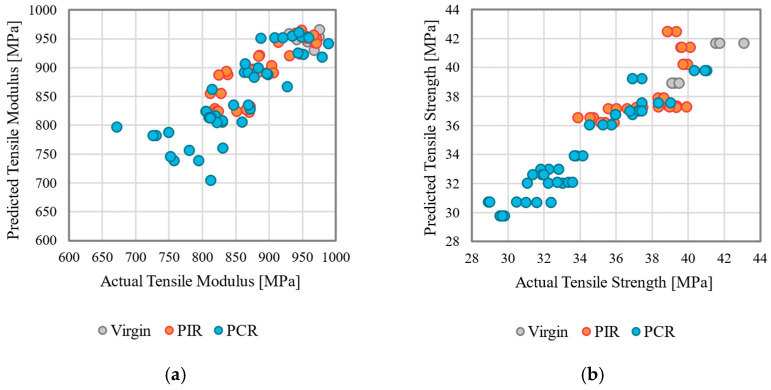
Visualization of the comparison of the determined and predicted mechanical properties of the film for the models with the highest R^2^: (**a**) tensile modulus, feature set 1, RF, (**b**) tensile strength, feature set 2, GAM.

In the second approach, modeling was conducted with CV to ensure the robustness and generalizability of the model (see Table 7 and Table 8). Comparing the model performance based on CV, feature sets 1 and 2 mostly demonstrated substantial improvements over the baseline dataset, with R^2^ values ranging from 67.77% (tensile modulus, feature set 1, RF) to 85.70% (tensile strength, FS2, and GAM) for both target variables. For feature set 0, the GAM model consistently outperformed LR and RF for the tensile modulus and strength across most evaluation metrics. The inclusion of the MFR in feature set 1 led to significant performance gains, as expected, due to strong correlations between MFR and the targets. The increase is most notable for LR in predicting tensile strength, where R^2^ increased from 6.52% to 47.15%. This improvement was also observed for RF in predicting the tensile modulus (R^2^ increase from 57.04% to 67.77%) (see Figure 5a).

Adding the viscosity gradient in feature set 2 resulted in marginal improvements for LR but did not enhance, and in some cases hindered, the performance of RF; for example, RF’s R^2^ for tensile strength prediction continuously decreased when MFR and viscosity gradient were added as input features. For the GAMs, the inclusion of the viscosity gradient increased model performance compared to feature sets 0 and 1, resulting in an R^2^ value of 85.7%, which can be observed in Figure 5b. Across all considered model-feature set-target combinations, the observed biases were predominantly negative, indicating a general tendency for the models to slightly underestimate the target. The MAEs ranged from 28.82 MPa (feature set 1, RF) to 52.09 MPa (feature set 0, LR) for tensile modulus, and 0.98 (feature set 2, LR) to 1.88 (feature set 0, LR) for tensile strength, further highlighting the substantial improvement achieved by incorporating the MFR along with the viscosity gradient in feature sets 1 and 2. Overall, the models demonstrated high predictive accuracy, as evidenced by consistently low MAPE (3.39–6.03% for tensile modulus and 2.83–5.37% for tensile strength). See Appendix C for additional visualizations.

## 6. Discussion

While data-driven approaches hold great promise for the prediction of properties, their effectiveness hinges on the availability of complete and detailed datasets encompassing formulation, processing, and material property information. The Generalized Additive, LR, and RF models used in this study show a high degree of accuracy and, even with the existing restrictions, a great potential for use in recyclate processing. Restrictions result from both modeling and recyclate processing. ML models depend not only on a high number of data points, but also on the quality and representativeness of the data. In this study, 28 material blends were examined, providing only a small database. By increasing the number of test series and examining more recyclates, modeling would be more significant and robust. With a larger database, it would also be possible to categorize batch fluctuations, which have a considerable influence on the quality and composition of a recyclate [52].

This also includes the different degradation caused by the contaminants present. The correlation between PIR and PCR compared to virgin material established in Section 5.2 is based on the area of application and the recyclate quality of the three feedstocks. The PIR materials came from an extrusion plant and are an extrusion grade. The PCR materials from the German near-household collection for PP are only available as an injection molding grade, which is generally characterized by a higher MFR compared to the film grade. An injection molding grade was also selected as virgin material due to its proximity to the recyclate. These correlations are therefore due to the different molecular structures of the materials, which lead to different viscosities. Due to the high MFR of an injection molding grade, the MFR is lowered when mixed with a high-viscosity film grade. In the case of pure virgin plastics, there is a correlation between the MFR and the mechanical properties of a film. Due to the unknown composition of the two materials PIR and PCR, plastic types, additives, or the molecular degradation of the materials can lead to different behavior and influences on the mechanical properties. In particular, the presence of impurities or gels in the film can be weak points in the film and impair the mechanical properties. Therefore, compared to virgin material, regardless of the MFR, a decrease in mechanical properties can be observed when using the recyclates of different feedstocks investigated here.

There was a striking correlation between the viscosity gradient and the mechanical properties of the material PIR2. There was a positive correlation for PIR1 and a negative correlation for PIR2. The viscosity values in Table 1 also show that the viscosity values were below those of the PP. This can be traced back to the recyclate quality of PIR2. This grade is originally a fully printed film, and these printing inks have a strong negative effect on the degradation [53,54], leading directly to a reduction in viscosity. This shows the importance of the material composition for the viscosity, which was not considered in this study.

Another important aspect is the combination of die pressure and viscosity parameters used in this study to model the mechanical properties. Die pressure is already used in most extrusion companies due to safety shutdowns of the extruder, resulting in a high potential of this measurement method. However, this study found that pressure measurement is influenced by many interference factors. These include, for example, the screw-in torque of the pressure sensor and the calibration of the pressure sensor. This can be seen by examining the results of the GAM without and with CV between feature sets 0 and 1. The existing systematic differences in the pressure of the two test series, which were not caused by the materials but by other effects such as the mounting of the sensor, resulted in deviations in the test data under otherwise constant measurement conditions. Standardization to the values of virgin PP was intended to reduce these effects, but it was not possible to prevent them completely. Due to the CV and the small amount of data, the distribution of the experimental data within the folds was not balanced, and the effect of the feature set was not as clear.

Thus, while data-driven models provide valuable insights into recyclate processing, their accuracy and reliability remain highly dependent on data quality, process stability, and material consistency. This highlights the need for further research and expanded datasets to improve predictive power and industrial applicability.

## 7. Conclusions and Outlook

In this study, the film extrusion process was modeled using die pressure during the processing of recyclates, thereby confirming research objective 1. Using Equation (1) and the associated correlation between pressure and viscosity, the various feedstocks can be characterized. The averaged pressures showed a dependence on the feedstock, with PCR materials showing low pressures, while PIR materials showed high pressures. Complementary measurements of shear viscosity and MFR characteristics showed a similar result. In our experiments, however, we also observed that the pressure can not only vary between test series with the same material but also be influenced by other interfering factors. These need to be identified and controlled in order to establish effective monitoring.

The mechanical properties, tensile strength and tensile modulus, were modeled and predicted using three combinations of the input parameters, namely, feedstock and pressure (feature set 0), feedstock, pressure, and MFR (feature set 1), and feedstock, pressure, MFR, and viscosity gradient (feature set 2). The modeling was carried out using LR, GAM, and RF, for which the coefficient of determination of the prediction was compared in each case. For the tensile modulus, the RF with feature set 1 approach yielded the most accurate predictions, with an R^2^ of 67.77%. In contrast, the tensile strength exhibited enhanced predictability, with an R^2^ of 85.70%, achieved by GAM on feature set 2. By considering material properties such as MFR and the viscosity gradient, batch effects could be accounted for, and the performance of the models was improved. These findings demonstrate the potential for predicting outcomes in recyclate processing by integrating material- and process-related parameters (research objective 2). We assume that the model performance can be further improved if differences in melt pressure between test series can be reduced and a larger amount of data is available, achieved by extended trials. However, further investigations are necessary to better characterize variation between batches of recyclates. For the RF model predicting tensile strength, expanding the feature set led to overfitting rather than performance gains. The resulting decrease in R^2^ from 77.92% with feature set 0 to 70.33% with feature set 2 suggests the model struggled to generalize, likely memorizing noise and performing poorly on unseen data. This tendency to overfit with inter-correlating data is a known issue for RF and can be mitigated by detailed hyperparameter tuning in future experiments.

Furthermore, viscosity is strongly dependent on the temperature. This influence was not considered in this study, so the use of an immersive melt temperature sensor is a useful addition. This will allow analyzing the influence of temperature in combination with pressure on viscosity and mechanical properties in the future. In a further step, it will also be tested if further material characteristics are suitable as input for the model. Recycled plastics show different melt enthalpies when compared with virgin material. It would be possible to characterize this using DSC measurements, but there is the challenge that the results can be falsified by impurities and the presence of other polymers [55,56].

As anticipated, this investigation into the impact of the feedstock on mechanical properties revealed, on average, the highest values for virgin materials. Irrespective of whether the recyclate is a PIR or PCR, the mechanical properties of the tensile test decrease with increasing recyclate content. To counteract this decline, the subsequent step should be to examine the influence of process parameters, such as the melt temperature. By varying the melt temperature, it is possible to influence the orientations introduced into the film and prevent decreasing mechanical properties due to the use of recyclate.

## Figures and Tables

**Table 1:** Rheological properties of the materials—MFR measured at 230 °C and shear viscosity at 220 °C.

Material	MFR [g/10 min]	Viscosity (41 1/s) [Pa·s]	Viscosity (82 1/s) [Pa·s]	Viscosity (204 1/s) [Pa·s]	Viscosity Gradient [-]
Virgin	7.50	584.5	411.2	237.6	−173.4
PIR1	3.94	723.7	479.6	270.6	−226.5
PIR2	5.48	505.2	355.1	221.5	−141.8
PCR1	16.72	399.0	299.0	185.9	−106.6
PCR2	13.00	467.4	337.4	202.0	−132.7
PCR3	12.50	508.2	334.5	206.1	−151.1

**Table 2:** Experimental plan for the production of the film samples.

Test ID	Share Virgin [%]	Share PIR1 [%]	Share PIR2 [%]	Share PCR1 [%]	Share PCR2 [%]	Share PCR3 [%]
T01	100	0	0	0	0	0
T02	75	25				
T03	50	50				
T04	25	75				
T05	0	100				
T06	75		25			
T07	50		50			
T08	25		75			
T09	0		100			
T10	75			25		
T11	50			50		
T12	25			75		
T13	0			100		
T14	75				25	
T15	50				50	
T16	25				75	
T17	0				100	
T18	8	92				
T19	50	50				
T20	69	31				
T21	8				92	
T22	50				50	
T23	69				31	
T24	0					100
T25	25					75
T26	50					50
T27	75					25
T28	100	0	0	0	0	0
Constant parameters						
Melt temper-ature [°C]	Kalander temper-ature [°C]	Film thickness [µm]	Film width [mm]	Mass through-put [kg/h]	Take-off speed [m/s]	
200	70	800	180	6.6	1.1	

**Table 3:** Process data measured in-line.

Material	Mean Pressure [bar]	Mean Torque [Nm]
Virgin	7.82	60.98
PIR1	10.86	66.40
PIR2	11.09	-
PCR1	6.71	-
PCR2	6.51	-
PCR3	5.52	-

**Table 4:** Overview of components used per feature set.

Feature Set 0 (FS0)	Feature Set 1 (FS1)	Feature Set 2 (FS2)
Feedstock + MFR	Feedstock + MFR + Pressure	Feedstock + MFR + Pressure + Viscosity gradient

**Table 5:** Correlation r values for both PIR and PCR types individually and combined (n = 84).

		PIR1	PIR2	PIR	PCR1	PCR2	PCR3	PCR
MFR [g/10 min]	Tensile Modulus	0.93	0.91	0.90	−0.96	−0.87	−0.94	−0.78
	Tensile Strength	0.92	−0.90	0.41	−0.99	−0.97	−0.99	−0.73
Pressure [bar]	Tensile Modulus	−0.68	−0.76	0.66	0.93	0.90	0.94	0.91
	Tensile Strength	−0.56	−0.75	0.56	0.97	0.96	0.98	0.94
Viscosity Gradient [-]	Tensile Modulus	0.93	−0.91	0.35	−0.96	−0.87	−0.94	−0.70
	Tensile Strength	0.92	−0.90	0.41	−0.99	−0.97	−0.99	−0.73

**Table 6:** Results for GAMs per feature set trained to predict tensile modulus and tensile strength.

	Tensile Modulus			Tensile Strength		
	FS0	FS1	FS2	FS0	FS1	FS2
ME [MPa]	0.02	0.02	0.01	0.00	0.00	0.00
SD [MPa]	34.29	30.40	27.01	1.02	0.80	0.62
MAE [MPa]	26.29	22.73	19.61	0.78	0.59	0.46
MAPE [%]	3.10	2.68	2.31	2.20	1.67	1.27
RMSE [MPa]	34.28	30.38	27.00	1.02	0.80	0.61
R^2^ [%]	78.70	83.27	86.79	91.56	94.74	96.91
OBJ [-]	33.89	28.98	24.95	0.94	0.72	0.54

**Table 7:** CV results for models per feature set trained to predict tensile modulus. Best R^2^ per feature set highlighted in bold.

	Tensile Modulus								
	FS0			FS1			FS2		
	GAM	LR	RF	GAM	LR	RF	GAM	LR	RF
ME [MPa]	−4.27	−7.08	0.65	−6.64	−4.50	−0.26	0.65	−1.09	2.77
SD [MPa]	46.28	57.02	41.79	49.64	38.85	34.19	45.83	29.07	35.07
MAE [MPa]	36.76	52.09	36.74	37.52	35.87	28.82	34.66	29.24	29.73
MAPE [%]	4.37	6.03	4.26	4.47	4.20	3.39	4.12	3.40	3.51
RMSE [MPa]	46.39	63.79	43.60	49.99	45.36	36.48	45.74	35.74	37.90
R^2^ [%]	61.00	6.52	57.04	54.71	47.15	67.77	62.08	67.22	66.43
OBJ [-]	36.49	19.96	20.25	35.60	38.22	14.91	30.21	27.52	14.86

**Table 8:** CV results for models per feature set trained to predict tensile strength. Best R^2^ per feature set highlighted in bold.

	Tensile Strength								
	FS0			FS1			FS2		
	GAM	LR	RF	GAM	LR	RF	GAM	LR	RF
ME [MPa]	−0.21	−0.28	0.03	−0.15	−0.17	0.03	−0.26	−0.03	0.08
SD [MPa]	1.52	2.10	1.40	1.56	1.46	1.46	1.3	0.93	1.49
MAE [MPa]	1.2	1.88	1.27	1.19	1.31	1.22	1.01	0.98	1.28
MAPE [%]	3.43	5.37	3.75	3.41	3.84	3.70	2.83	2.88	3.85
RMSE [MPa]	1.53	2.38	1.48	1.56	1.73	1.59	1.32	1.21	1.63
R^2^ [%]	80.75	36.64	77.92	80.08	66.51	72.56	85.7	83.47	70.33
OBJ [-]	1.01	2.69	0.64	0.82	1.25	0.55	0.68	0.91	0.57

**Figure A1:**
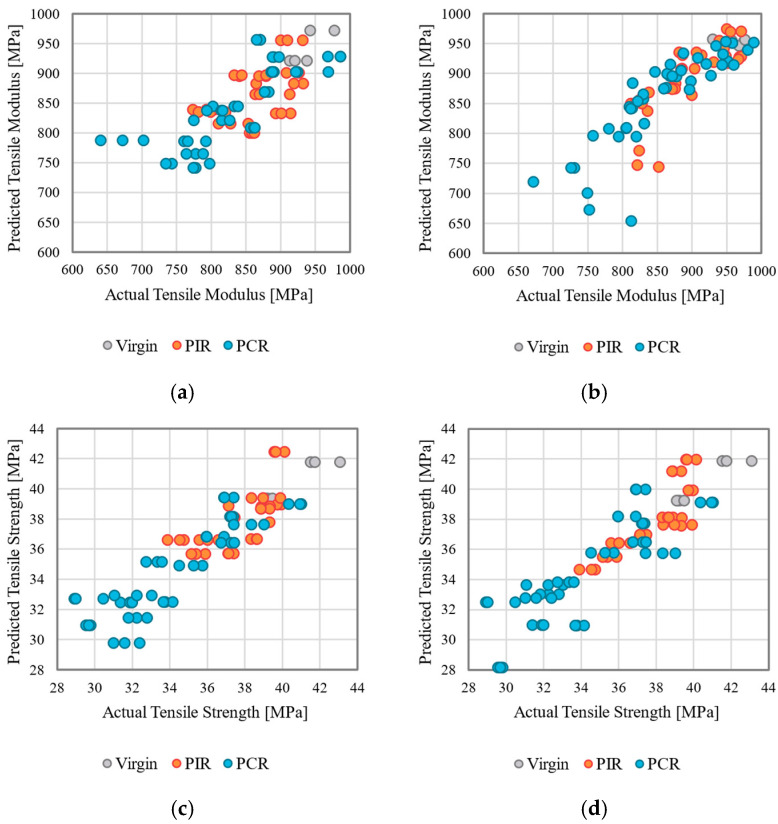
Visualization of the comparison of the determined and predicted mechanical properties of the film for the models with the highest R^2^: (**a**) tensile modulus FS0 GAM, (**b**) tensile modulus FS2 LR, (**c**) tensile strength FS0 GAM, (**d**) tensile strength FS1 GAM.

## Data Availability

The original contributions presented in this study are included in the article. Further inquiries can be directed to the corresponding author.

## Appendix A. Measures of Location and Variation of Pressure Records During Film Extrusion in Bar for Each Test

**Table:**

Test ID	Mean	Std	Min	25%	50%	75%	Max
T01	8.52	0.46	7.6	8.19	8.55	8.93	9.31
T02	9.64	0.38	8.83	9.39	9.62	9.93	10.44
T03	10.34	0.43	9.26	10.08	10.33	10.64	11.25
T04	11.06	0.43	10.08	10.77	11.02	11.38	12.09
T05	11.36	0.4	10.44	11.15	11.35	11.66	12.14
T06	9.79	0.54	8.57	9.44	9.82	10.21	10.87
T07	10.7	0.57	9.39	10.31	10.74	11.1	11.86
T08	11.72	0.63	10.54	11.25	11.63	12.25	13.06
T09	12.14	0.55	11.05	11.71	12.12	12.53	13.42
T10	7.78	0.32	7.14	7.53	7.73	7.99	8.7
T11	7.14	0.27	6.56	6.94	7.14	7.32	7.81
T12	6.39	0.24	5.87	6.23	6.38	6.56	6.99
T13	5.52	0.19	5.18	5.36	5.46	5.66	6.02
T14	7.97	0.36	7.27	7.71	7.94	8.27	8.75
T15	7.23	0.21	6.79	7.04	7.22	7.37	7.76
T16	6.5	0.22	6.05	6.33	6.48	6.69	7.02
T17	5.79	0.19	5.49	5.61	5.74	5.95	6.28
T18	13.39	0.49	12.15	13.06	13.32	13.73	17.71
T19	11.55	0.47	10.05	11.18	11.48	11.84	15.31
T20	8.7	0.3	7.35	8.49	8.7	8.9	10.66
T21	5.15	0.27	4.17	4.98	5.1	5.27	7.55
T22	6.15	0.28	5.39	5.96	6.12	6.29	9.43
T23	6.77	0.28	5.76	6.61	6.78	6.9	9.76
T24	4.58	0.23	3.76	4.45	4.57	4.7	7.88
T25	5.26	0.26	4.08	5.1	5.23	5.39	7.8
T26	5.85	0.28	4.29	5.68	5.84	6	8.82
T27	6.39	0.26	5.63	6.21	6.37	6.53	7.96
T28	7.12	0.31	6.25	6.9	7.1	7.31	8.9

## Appendix B. Equations for Calculating the Measures of Determination

$$MAE\;=\;\frac{1}{n}\;\sum \left|y_{i}\;-{\hat{y}}_{i}\right|$$

$$OBJ\;=\;\left(\frac{n_{train}}{n_{all}}\right)\;\frac{RMSE_{train}\;+\;MAE_{train}}{R_{train}^{2}\;+\;1}\;+\;\left(\frac{n_{test}}{n_{all}}\right)\;\frac{RMSE_{test}\;+\;MAE_{test}}{R_{test}^{2}\;+\;1}$$

$$MAPE\;=\;\frac{1}{n}\sum \left|\frac{y_{i}\;-\;{\hat{y}}_{i}}{y_{i}}\right|$$

$$RMSE\;=\sqrt{\;\frac{1}{n}\sum {\left(y_{i}\;-\;{\hat{y}}_{i}\right)}^{2}}$$

$$r\;=\;\frac{\sum \left(x_{i}\;-\;\bar{x}\right)\left(y_{i}\;-\;\bar{y}\right)}{\sqrt{\sum {\left(x_{i}\;-\;\overset{-}{x}\right)}^{2}\;\sum {\left(y_{i}\;-\;\overset{-}{y}\right)}^{2}}}$$

$$R2\;=\;1\;-\;\frac{\sum {\left(y_{i}\;-\;{\hat{y}}_{i}\right)}^{2}}{\sum {\left(y_{i}\;-\;{\overset{-}{y}}_{i}\right)}^{2}}$$
